# Exploring the composition of placental microbiome and its potential origin in preterm birth

**DOI:** 10.3389/fcimb.2024.1486409

**Published:** 2025-01-16

**Authors:** Marwa Saadaoui, Mohamed Nadhir Djekidel, Selvasankar Murugesan, Manoj Kumar, Duaa Elhag, Parul Singh, Basirudeen Syed Ahamed Kabeer, Alexandra Katharina Marr, Tomoshige Kino, Tobias Brummaier, Rose McGready, François Nosten, Damien Chaussabel, Annalisa Terranegra, Souhaila Al Khodor

**Affiliations:** ^1^ Research Department, Sidra Medicine, Doha, Qatar; ^2^ Center for Global Health Research, Saveetha Medical College and Hospital, Saveetha University, Chennai, India; ^3^ Shoklo Malaria Research Unit, Mahidol-Oxford Tropical Medicine Research Unit, Faculty of Tropical Medicine, Mahidol University, Mae Sot, Thailand; ^4^ Centre for Tropical Medicine and Global Health, Nuffield Department of Medicine, University of Oxford, Oxford, United Kingdom; ^5^ The Jackson Laboratory for Genomic Medicine, Farmington, CT, United States

**Keywords:** pregnancy complications, microbiota, saliva, placenta, premature delivery

## Abstract

**Introduction:**

For years, the placenta was believed to be sterile, but recent studies reveal it hosts a unique microbiome. Despite these findings, significant questions remain about the origins of the placental microbiome and its effects on pregnancy and fetal health. Some studies suggest it may originate from the vaginal tract, while others indicate that oral bacteria can enter the maternal bloodstream and seed the placenta. However, research analyzing the vaginal, oral, and placental microbiomes within the same cohort is lacking. Additionally, it’s unclear whether the placental microbiome differs between healthy pregnancies and those with complications like preterm birth (PTB), which remains a leading cause of neonatal morbidity and mortality worldwide.

**Methods:**

In this study, we performed 16S rRNA gene sequencing to investigate the composition of the oral and placental microbiome in samples collected from 18 women who experienced PTB and 36 matched controls who delivered at term (TB), all of whom were part of the Molecular Signature in Pregnancy (MSP) study. We leveraged on the multisite microbiome sampling from the MSP participants and on our previously published vaginal microbiome data to investigate the potential origins of the placental microbiome and assess whether its composition varies between healthy and complicated pregnancies.

**Results and Discussion:**

Our analysis revealed distinct profiles in the oral microbiome of PTB subjects compared to those who delivered at term. Specifically, we observed an increased abundance of Treponema maltophilum, Bacteroides sp, Mollicutes, Prevotella buccae, Leptotrichia, Prevotella_sp_Alloprevotella, in the PTB group. Importantly, Treponema maltophilum species showed higher abundance in the PTB group during the second trimester, suggesting its potential use as biomarkers. When we assessed the placenta microbiome composition, we found that Firmicutes, Bacteroidetes, Actinobacteria, and Proteobacteria were the most dominant phyla. Interestingly, microorganisms such as Ureaplasma urealyticum were more abundant in PTB placenta samples. Our findings suggest that the placenta microbiome could originate from the oral or vaginal cavities, with a notable increase in the crosstalk between the vaginal and placental sites in cases of PTB. Specifically, our data revealed that in PTB cases, the placental microbiome exhibited a closer resemblance to the vaginal microbiome, whereas in term pregnancies, the placental microbiome was similar to the oral microbiome.

## Introduction

Preterm birth (PTB), known as childbirth before completing 37 weeks of gestation, is a major cause of neonatal health issues and mortality worldwide (Quinn et al., 2016). Each year, approximately 15 million cases are reported globally, with over 50% occurring in Asia, highlighting PTB as a critical public health challenge (Chawanpaiboon et al., 2019; Walani, 2020). The causes of PTB are complex and not fully understood (Luk et al., 2023). Key risk factors include a history of PTB, infections in the genitourinary tract, reduced progesterone levels, shorter cervical length, maternal stress, ethnicity, and body mass index (Green and Arck, 2020).

Pregnancy represents a distinct phase in a woman’s life, that is characterized by significant physiological adaptations required to create an optimal environment for fetal development (Prince et al., 2015; Soma-Pillay et al., 2016). These changes extend to the body’s microbial communities, collectively referred to as the microbiota, which includes bacteria, fungi, and viruses that inhabit various environments on and within the body (Walker et al., 2015). Throughout pregnancy, the composition and abundance of the maternal microbiome undergoes dynamic shifts to maintain balance and support fetal growth (Koren et al., 2012; Nunn et al., 2021; Ye and Kapila, 2021; Gorczyca et al., 2022; Zakaria et al., 2022; Giannella et al., 2023; Li et al., 2024).

For years, it was believed that the placenta is a sterile environment (Aquino et al., 1984; Perez-Munoz et al., 2017). However, many studies have detected bacteria in the placenta (Pankuch et al., 1984; Hillier et al., 1991; Baud and Greub, 2011; Whidbey et al., 2013; Aagaard et al., 2014; Kim et al., 2015; Arora et al., 2017; Cappelletti et al., 2020). Others that used 16S rRNA and whole-genome shotgun gene-sequencing technologies (Aagaard et al., 2014; Collado et al., 2016; Zheng et al., 2017; La et al., 2022) have revealed that the placenta has a unique microbiome dominated by four major phyla: Firmicutes, Bacteroidetes, Proteobacteria and Actinobacteria (Stout et al., 2013; Aagaard et al., 2014; Al-Kaabi and Atherton, 2015; Collado et al., 2016; Yang et al., 2024). Notably, recent investigations have reported the presence of a microbiota in placentas from uncomplicated pregnancies at term (Aagaard et al., 2014; Collado et al., 2016; Gomez-Arango et al., 2017; Seferovic et al., 2019), and have concluded that the bacterial profiles of placentas from pregnancies complicated by spontaneous PTB (Aagaard et al., 2014), gestational diabetes mellitus (Bassols et al., 2016) and severe chorioamnionitis (Prince et al., 2016) differ from those of placentas from uncomplicated pregnancies at term. It’s important to consider that changes in the placental microbiome during chorioamnionitis might be influenced by potential contamination from ascending vaginal bacteria (Yin et al., 2023). The risk of chorioamnionitis increases with prolonged rupture of membranes (over 18 hours), which may allow microbes to invade the placenta and amniotic cavity (Yin et al., 2023).

The origin of the placental microbiome remains unclear, with some studies proposing the placental microbiome to originate from the vagina while others providing evidence of an oral source (Han et al., 2004; Vander Haar et al., 2018; Amir et al., 2020; Olaniyi et al., 2020; Fan et al., 2023; Xiao and Zhao, 2023). Studies investigating the interrelationships among oral, vaginal, and placental microbiomes remain sparse, and whether those interrelationships differ between pregnant women who deliver at term and those who experience pregnancy complications such as PTB remains largely unknown. In a previous study, we characterized the vaginal microbiome of women of Karen and Burman ethnicity enrolled in the Molecular Signature in Pregnancy (MSP) cohort, and identified a predictive vaginal microbiome signature for PTB, characterized by higher levels of *Prevotella buccalis*, and lower levels of *Lactobacillus crispatus and Finegoldia* (Kumar M et al., 2020). We also showed that this signature was detectable as early as in the first trimester of pregnancy (Kumar M et al., 2020). Leveraging on this prospective, high frequency, multi-site sampling cohort, we aim to characterize the oral and placental microbiome in the MSP study subjects and assess the interrelationship of the oral, vaginal, and placental microbiomes in pregnant women who delivered at term and compare it to those who experienced PTB.

The oral microbiome is composed of approximately 700 species (Deo and Deshmukh, 2019; Saadaoui et al., 2021), including *Streptococci, Lactobacilli, Staphylococci*, and *Corynebacteria* (Butera et al., 2021). Various environmental factors, such as pH, anaerobic conditions, diet, hormonal fluctuations, and access to a dentist which is largely absent in low-resource settings, can influence the richness and composition of the oral microbiome (Sedghi et al., 2021; Saadaoui et al., 2021). During pregnancy, hormonal, immunological, and physiological changes can lead to increased risk for oral diseases, such as periodontal disease and dental caries (Ressler-Maerlender et al., 2005; Silva de Araujo Figueiredo et al., 2017; Saadaoui et al., 2021). Many studies have identified periodontal disease as a potential risk factor for PTB (Bansal et al., 2011; Cetin et al., 2012; Zi et al., 2014; Uwitonze et al., 2018; Komine-Aizawa et al., 2019; Figuero et al., 2020; Isola et al., 2020; Ye et al., 2020; Pockpa et al., 2021; Alnasser et al., 2023; Bobetsis et al., 2023) and showed that the rates of PTB increase with the severity of periodontitis and gingivitis (Marquez-Corona et al., 2021). Others have shown that the levels of *Porphyromonas gingivalis*, *Fusobacterium nucleatum, Treponema denticola, and Aggregatibacter actinomycetemcomitans* in the oral cavity was significantly higher in PTB subjects compared to those who delivered full term (Ye et al., 2013; Ye et al., 2020; Jang et al., 2021). Transmission of oral bacteria to the placenta can occur through the bloodstream (Fardini et al., 2010), with *F. nucleatum* detected in the dental plaques, placenta, and amniotic fluid of up to 30 percent of women delivering preterm (Han et al., 2004; Lima et al., 2023). Periodontitis has also been strongly associated with low birth weight (LBW) in newborns (Heo et al., 2020). Pregnant women with high levels of cavity-causing bacteria may transfer these bacteria to their babies’ mouths after delivery (Ramos-Gomez et al., 2010; Ramos-Gomez et al., 2010). It is worth noting that dental caries and periodontal disease in pregnant women can be prevented, yet efforts to improve oral healthcare during pregnancy are still limited especially in lower income countries (Peres et al., 2019; Bawaskar and Bawaskar, 2020).

The purpose of this study is to characterize the oral and placental microbiome in samples collected from a low-resource setting in women of Karen and Burman ethnicity who delivered prematurely compared to matching controls who delivered full term. We will also shed some light on the potential source of the placental microbiome. To our knowledge, this is the first study investigating the origin of the placental microbiome and the interrelationship between the various microbiomes in pregnant women who delivered at term and compare it to those who experienced preterm birth.

## Materials and methods

### Study design

This observational, prospective pregnancy/delivery postpartum cohort study was conducted as a collaboration between Sidra Medicine, Doha, Qatar, and the Shoklo Malaria Research Unit (SMRU), Mae Sot, Thailand (Brummaier et al., 2019). SMRU is a field station of the Faculty of Tropical Medicine, Mahidol University, Bangkok, Thailand. The collaboration aims to improve the lives of rural and disadvantaged migrant and refugee populations residing on the Thailand-Myanmar border by combining research with humanitarian efforts. This research project was approved by the Ethics Committee of the Faculty of Tropical Medicine, Mahidol University in Bangkok, Thailand (Ethics Reference: TMEC 15-062), the Oxford Tropical Research Ethics Committee (Ethics Reference: OxTREC: 33-15), the Institutional Review board (IRB) at Sidra Medicine (Protocol#1705010909). The study was carried out in accordance with the ethical principles outlined in the Declaration of Helsinki and followed the ICH Guidelines for Good Clinical Practice.

### Participant enrollment and clinical history

First-trimester (T1) pregnant women with a viable, singleton pregnancy were enrolled at SMRU’s antenatal care clinics located on the Thailand–Myanmar border (Brummaier et al., 2019). The gestational age of the pregnancy was determined using early ultrasound scans. Women between the ages of 18 and 49 years, with an estimated gestational age ranging from 8 weeks 0 days to 13 weeks 6 days at the time of enrollment, were invited to participate in the study. The processes of enrolling pregnant women and collecting samples, including the criteria for inclusion and exclusion, have been described in detail in a previous publication (Brummaier et al., 2019). At enrolment, comprehensive maternal demographic information, medical and obstetric history were recorded. Additionally, a thorough physical and obstetric examination was conducted. Participants in the MSP study consented to high-frequency blood sample collection, multi-site sampling for microbiome analysis, including stool, saliva, vaginal swabs and placenta samples obtained during pregnancy, delivery and post-partum periods (Brummaier et al., 2019). Stool samples and vaginal swabs were collected during each trimester and at delivery, while saliva samples were collected in the second trimester and at delivery (**Supplementary Figure 1**). Placenta samples were collected at the time of delivery (**Supplementary Figure 1**). As part of the MSP cohort, 19 participants experienced PTB (Kumar M et al., 2020). One PTB subject was excluded due to insufficient sample availability. A total of 54 pregnant women were included in this study with 18 PTB subjects and 36 matching controls. The case-control matching of the participants was performed as previously described based on age, parity, and gravidity (Kumar M et al., 2020).

### Sample collection

#### A) Saliva sampling

Saliva samples were collected at two time points: at 24-26 weeks of gestation and at delivery. The samples were taken at least 30 minutes after the participant’s last food intake. Prior to sample collection, each participant was asked to rinse her mouth with clean water for at least 30 seconds. Then, the participant spat approximately 3 ml of saliva into a sterile falcon tube. Two aliquots of 0.5 ml each were transferred into sterile Eppendorf tubes and stored without further processing. Additionally, two 0.5 ml aliquots were transferred into sterile Eppendorf tubes and mixed with 0.5 ml of RNAlater. All saliva samples were stored at −80°C before processing.

#### B) Placental tissue sampling

Placenta samples were taken and processed within 30 minutes of placental expulsion. Sterile techniques were applied to harvest placental tissue. Healthy placenta tissue located 3 cm from the edge of the placenta was identified. A rectangle measuring 0.5 cm across and 3 cm in length and approximately 1- to 1.5-cm deep, was cut from the maternal surface while avoiding cutting through the membranes covering the fetal side. Afterwards, 0.25-0.5 cm of the maternal surface of the placenta was removed, and 9 cubes measuring 0.5 x 0.5 x 0.5 cm each were excised. All samples were rinsed in sterile phosphate-buffered saline, then transferred into cryovials and stored in liquid Nitrogen.

#### C) Vaginal swab collection

As previously described (Kumar M et al., 2020), vaginal swabs from the posterior fornix were collected during the first trimester (8 weeks 0 days to 13 weeks 6 days, second trimester (20-24 weeks), and third trimester of pregnancy (32-35 weeks) as well as at the time of delivery. Vaginal swabs were collected using the Copan Eswab™ collection system. Samples were stored at -80°C before processing.

### DNA isolation and 16S rRNA gene sequencing

DNA was extracted from vaginal swab, saliva and placental tissue using the modified MoBio Powersoil as previously reported (Mattei et al., 2019). Then DNA was quantified using Nanodrop, and the V1-V3 regions of the 16S rDNA were amplified using 27F forward primers attached to a 12-bp specific Illumina 5′ adapter to provide barcodes for each sample in addition to the common reverse primer 515 R (Mattei et al., 2019). In brief, PCR was applied in triplicate using a 50-ml reaction mixture containing 10 ng of template DNA and 2x Phusion HotStart Ready Mix. The following protocol was used for thermal cycling: 5 min of primary denaturation at 94°C; 25 cycles of denaturation at 94°C for 30 s, annealing at 62°C for 30 s, elongation at 72°C for 30 s; and an end step of 72°C for 10 min. The 650-bp amplified PCR products from each saliva or placenta sample were respectively pooled in equimolar concentrations. Pooled PCR products were purified utilizing AgenCourt AMPure XP magnetic beads. High-throughput sequencing was applied on an Illumina MiSeq 2 × 300 platform (Illumina, Inc., San Diego, CA, USA) according to the manufacturer’s instructions. Image analysis and base calling were both performed on MiSeq.

### Microbiome data analysis

Raw reads from vaginal swab, saliva and placenta samples were processed using the standard Qiim2 + dada2 pipeline (Canavese et al., 1980). The “qiime cutadapt” command was used to trim V1-V3 Adapter sequences (V1_F: AGAGTTTGATCMTGGCTCAG, V3_R: GWATTACCGCGGCKGCTG). Down-stream analysis was mainly achieved using the MicrobiotaProcess R/Bioconductor package (v) (Xu et al., 2023). To account for biases in sequencing depth, we rarified the amplicon sequence variant count tables to 10,000 reads per sample. The sequencing depth used for rarefication was based on the alpha rarefication curves to ensure a sufficient representation of the microbial community. The ASV count data were normalized using the total sum and scaling for relative abundance at the phylum and genus level was completed using the “mp_decostand” function. Principal coordinate analysis (PCoA) ordination on the combined tissue data was completed using weighted-unifrac distances (after Hellinger transformation) at the delivery timepoint. The permutational multivariate analysis of variance test Adonis was used to assess the statistical significance of the clustering of samples. The Zicoseq method (Yang and Chen, 2022) was used to detect differentially abundant species.

### Placental microbiome source tracking and estimation of bacterial sharing between different body sites and the placenta

To understand the source(s) of the placental microbiome and to estimate the extent of microbial sharing between different body sites and placenta, we used the fast expectation-maximization for microbial source tracking (FEAST) (v0.1.0) (Shenhav et al., 2019). Only pregnant women with available samples collected from the placenta and the two other body sites at the delivery time point were considered in this analysis. To get more reliable results, we only considered samples that have a sequencing depth of at least 5,000 reads. Additionally, FEAST was run using 1,000 expectation-maximization (EM) iterations. In this analysis, we included previously published vaginal microbiome sequencing data (Kumar M et al., 2020). The tissue contribution was calculated as the individual level. The average sharing (shown in the pie chart) was scaled to 100%. A bacterial species was categorized as “shared” if it was detected in a sample-placenta pair from the same pregnant women. The percentage of shared microbial species was calculated as the proportion of subjects with shared species out of the total number of subjects evaluated at that time point.

### Plotting and statistical analysis

All downstream analyses were done using R language (v4.3.1). Statistical tests were calculated using the rstatix package (v0.7.2) (https://rpkgs.datanovia.com/rstatix/index.html). Plots were generated using ggplot2 (v3.4.4) and ComplexHeatmap packages (v2.15.4) (Gu et al., 2016; Gu, 2022).

### Dysbiosis score calculation

To access the microbial community disruption in PTB samples, we calculated the dysbiosis score using the dysbiosisR package. For each time point in each tissue, unifrac distances were calculated to capture the differences between microbial communities. The dysbiosis score was then calculated using the dysbiosisMedianCLV function and using the TB samples as reference. The statistical significance of dysbiosis score between groups was estimated using Wilcoxon rank-sum tests. To estimate the correlation between the dysbiosis score for each tissue at each time point, we did a Spearman’s correlation test using the cor.test function.

## Results

### Description of the cohort

To investigate the microbiome composition in PTB subjects, we designed a nested case-control study involving 18 PTB cases and 36 TB controls matched for key demographic, anthropometric and clinical variables (**Supplementary Table 1**) (Kumar M et al., 2020). There were no significant differences in maternal age, height, weight, body mass index, mode of delivery or in the length of the rupture of membranes between the PTB and TB groups (**Supplementary Table 1**) (Kumar M et al., 2020). The average gestational age at delivery for the PTB cases was 36.2 weeks, whereas for TB controls, it was 39.5 weeks, with PTB neonates exhibiting lower birth weights as anticipated (**Supplementary Table 1**) (Kumar M et al., 2020). Type and number of samples collected at various time points from TB and PTB subjects are summarized in **Supplementary Figure 1**.

### Composition of the maternal microbiome varies during pregnancy and in women with PTB

In our previous study, using 16S ribosomal RNA gene sequencing we assessed the vaginal microbiome composition in 18 PTB subjects compared to 36 matching controls who delivered at term (Kumar M et al., 2020). Our findings revealed a predictive vaginal microbiota signature for PTB detectable as early as the first trimester of pregnancy (Kumar M et al., 2020). This signature featured elevated levels of *Prevotella buccalis* and reduced levels of *Lactobacillus crispatus* and *Finegoldia* (Kumar M et al., 2020).

**Figure 1:**
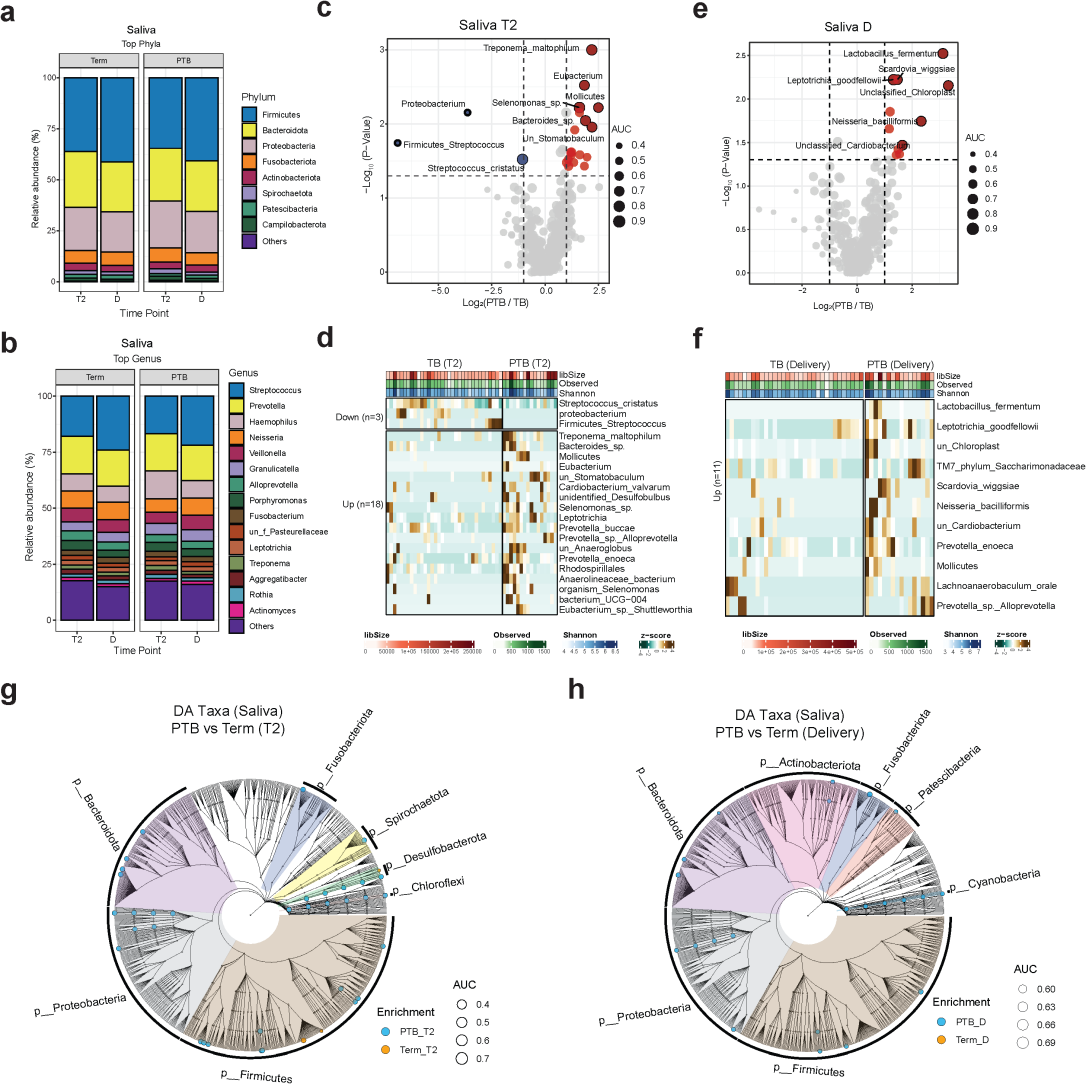
Oral microbiome composition. **(A, B)** Stacked bar charts showing the average relative abundance (%) of the 15 most enriched phyla **(A)** and genera **(B)** in PTB and TB women. Each vertical bar represents one timepoint (T2 or D). **(C, E)** Volcano plot showing the log2(FC) and the *p*-values of the differentially abundant species at T2 and delivery. Red: enriched in PTB; blue: enriched in TB. **(D, F)** Heatmap showing the z-scores for the relative abundance of differentially abundant oral species at T2 and delivery. Brown: high enrichment; dark green: low enrichment. **(G, H)** Cladograms showing the differentially abundant taxa at different taxonomical levels grouped by phylum at T2 and delivery (criteria: FC ≥2 and *p* value < 0.05). Blue dots: enriched in PTB; orange dots: enriched in TB. Trimester (T), delivery **(D)**, TB (Term birth), PTB (Preterm birth).

In this paper, we investigate the microbiome composition of saliva and placenta samples collected from the same cohort of TB and PTB subjects previously studied for vaginal microbiota composition (Kumar M et al., 2020). First, we conducted a comparison of the salivary microbiome composition between the second trimester (T2) and the time of delivery among 54 pregnant women who experienced PTB or TB. Our aim was to investigate the differences in microbial richness, diversity and identify differentially abundant taxa. In terms of microbial composition across all pregnant women, Firmicutes, Bacteroides, Proteobacteria, and Fusobacteria were the dominant phyla in the saliva samples, collectively constituting over 80% of the total microbial abundance (**Figure 1A**). Our analyses identified the presence of 7,850 amplicon sequence variant (ASV) that could, after removal of rare sequences be assigned to 154 known taxa to the level of genera (**Figure 1B**). At the genus level, the most prevalent genera observed in saliva samples included *Streptococcus*, *Prevotella*, *Haemophilus*, *Neisseria*, and *Veillonella* among others (**Figure 1B**).

To identify the most abundant species at the second trimester, we conducted differential abundance analysis (**Figures 1C, D**; **Supplementary Table 2**). A total of 18 species including *Treponema maltophilum*, *Bacteroides sp, Mollicutes*, *Prevotella buccae*, *Leptotrichia*, *Prevotella_sp_Alloprevotella*, unclassified *Anaeroglobus*, among others were more abundant in the PTB than the TB group at T2 (*p*-value < 0.05 and FC ≥2). Whereas *Streptococcus cristatus* were the most abundant species in the TB group (**Figures 1C, D**; **Supplementary Table 2**).

At delivery, we observed an increase in the abundances of several species in the PTB group, including *Prevotella enoeca*, *Lachnoanaerobaculum*_oral, *Leptotrihia goodfellowi*, TM7, *Prevotella*_sp_*Alloprevoella*, unclassified *Cardiobacterium*, *Neisseria bacilliformis*, and *Lactobacillus fermentum* (**Figures 1E, F**; **Supplementary Table 2**). We then ran a differential abundance analysis at different taxonomical levels and generated a cladogram to compare the differences in the salivary microbiome at T2 and delivery to get a global overview of microbial community changes (**Figures 1G, H**). Our data shows that most of the salivary microbiome compositional changes between PTB and TB was observed at the second trimester rather than at delivery.

To assess the microbial diversity and community structure within PTB and TB saliva samples, we conducted alpha and beta diversity analyses (**Supplementary Figures 2A-C**). None of the alpha diversity indices used, including Chao1, observed operational taxonomic units (OTUs), Shannon, and Simpson indicated statistically significant differences (**Supplementary Figure 2A**). On the other hand, beta diversity measures calculated using Bray–Curtis distance metrics showed a significant difference in the salivary microbiome composition when the TB and PTB groups were compared (*p*= 0.001) but not when we compared the diversity within the different time points (**Supplementary Figures 2B, C**).

**Figure 2:**
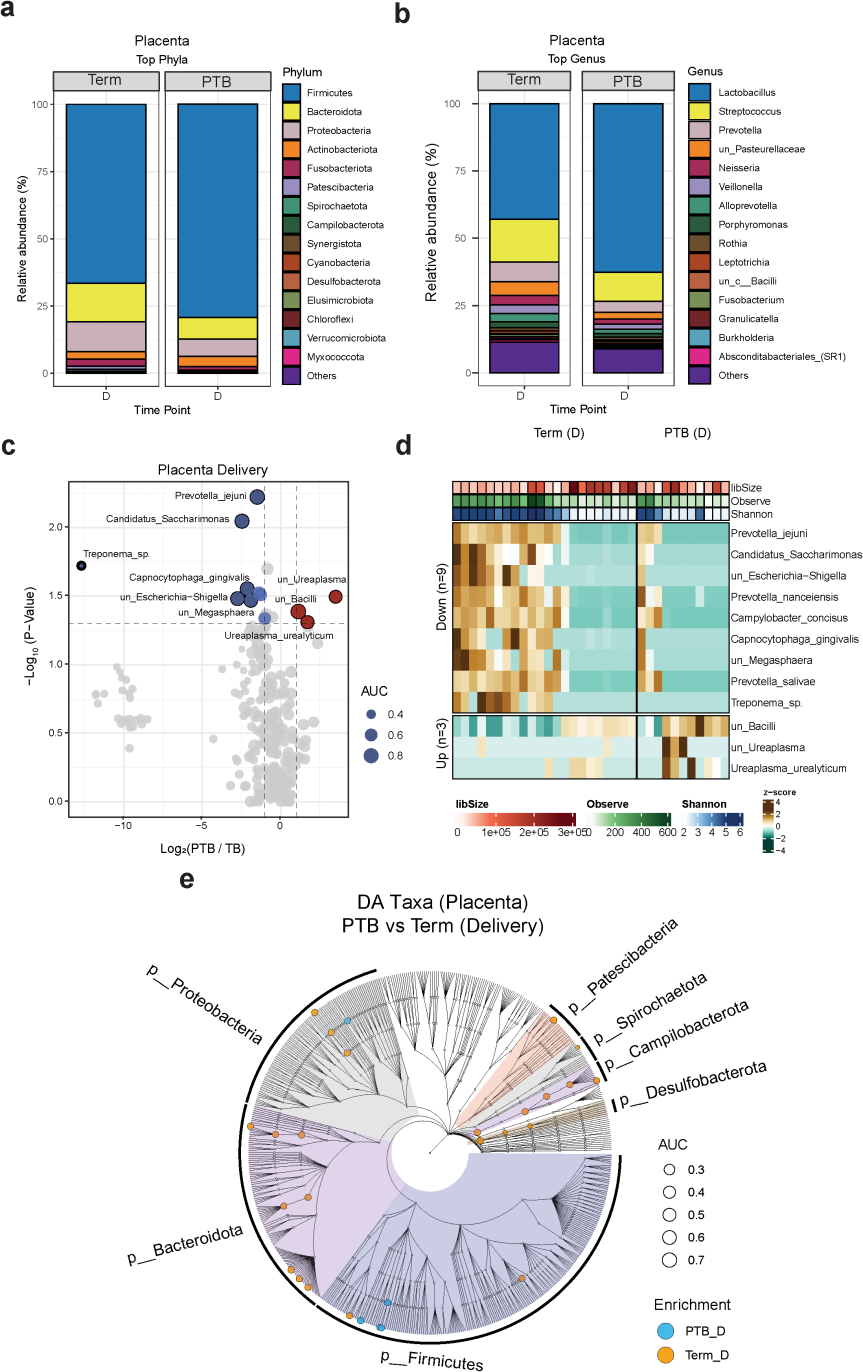
Placental microbiome composition. **(A, B)** Stacked bar charts showing the average relative abundance (%) of the 15 most enriched phyla **(A)** and genera **(B)** in PTB and TB women at delivery. **(C)** Volcano plot showing the log2(FC) and *p*-values of differentially abundant species during delivery. Red: enriched in PTB; blue: enriched in TB. **(D)** Boxplot showing the relative abundance distribution of the differentially abundant species between TB and PTB samples. Green: PTB; purple: TB. **(E)** Cladograms showing the differentially abundant taxa at different taxonomical levels grouped by phylum at delivery (criteria: FC ≥2 and *p* value < 0.05). Blue dots: enriched in PTB; orange dots: enriched in TB.

We next compared the placental microbiome composition in the study cohort. To rule out the possibility of contamination, we run water samples as negative controls (**Supplementary Figure 3**), and to exclude potential bacterial contamination from membrane rupture, we removed samples from subjects who experienced membrane rupture lasting longer than 18 hours (3 TB and 3 PTB subjects). Our data showed that Firmicutes, Bacteroidota, Proteobacteria, and Actinobacteria were the most abundant phyla observed in all placenta samples, covering approximately 90% of total microbial abundance (**Figure 2A**). At the genus level, the most prevalent genera detected in the placenta samples were *Lactobacillus*, *Streptococcus*, *Prevotella*, *Neisseria*, and *Veillonella* (**Figure 2B**). We then conducted additional taxonomic analysis at the species level (**Figures 2C, D**). We observed that *Ureaplasma urealyticum* and *Ureaplasma species* were more abundant in the PTB group, while *Candidatus saccharimonas, Prevotella jejuni, Capnocytophaga gingivalis and Megasphaera* sp. were more abundant in the TB group (**Figures 2C, D**). Globally, at all taxonomic levels, the cladogram showed that the TB group had a higher richness of taxa compared to the PTB group (**Figure 2E**).

To assess the microbial diversity and community structure in PTB and TB placenta samples, we conducted alpha and beta diversity analyses, but we did not observe any significant differences within both groups (**Supplementary Figures 2D, E**).

Finally, we assessed the dysbiosis score in saliva, vaginal, and placental samples from both PTB and TB groups (**Supplementary Figure 4**). We found that the dysbiosis score was significantly higher in PTB samples compared to TB samples during the second and third trimesters, specifically in oral and vaginal samples, respectively. The dysbiosis score was positively correlated with the observed species index suggesting that an increase in microbial richness in vaginal and saliva samples can lead to dysbiosis, however an opposite pattern was observed in the placenta, where a higher dysbiosis score was inversely correlated with microbial richness, indicating that the loss of diversity and dominance of few microbial species may be the main cause of dysbiosis (**Supplementary Figure 5**).

### Exploring the potential origin of the placental microbiome: role of the vaginal and oral microbiome

To explore the possible origin of the placental microbiome and assess the interrelationship between the various microbiomes in pregnant women who delivered at term and compare it to those who experienced PTB, we used FEAST (Shenhav et al., 2019). This algorithm takes as input a data set of microbial communities containing the “sink” (placenta) and a separate group of potential “sources” (vagina and oral sites), and then quantifies the fraction of each source and unknown origins including contaminants contribution in the sink community (Shenhav et al., 2019).

**Figure 3:**
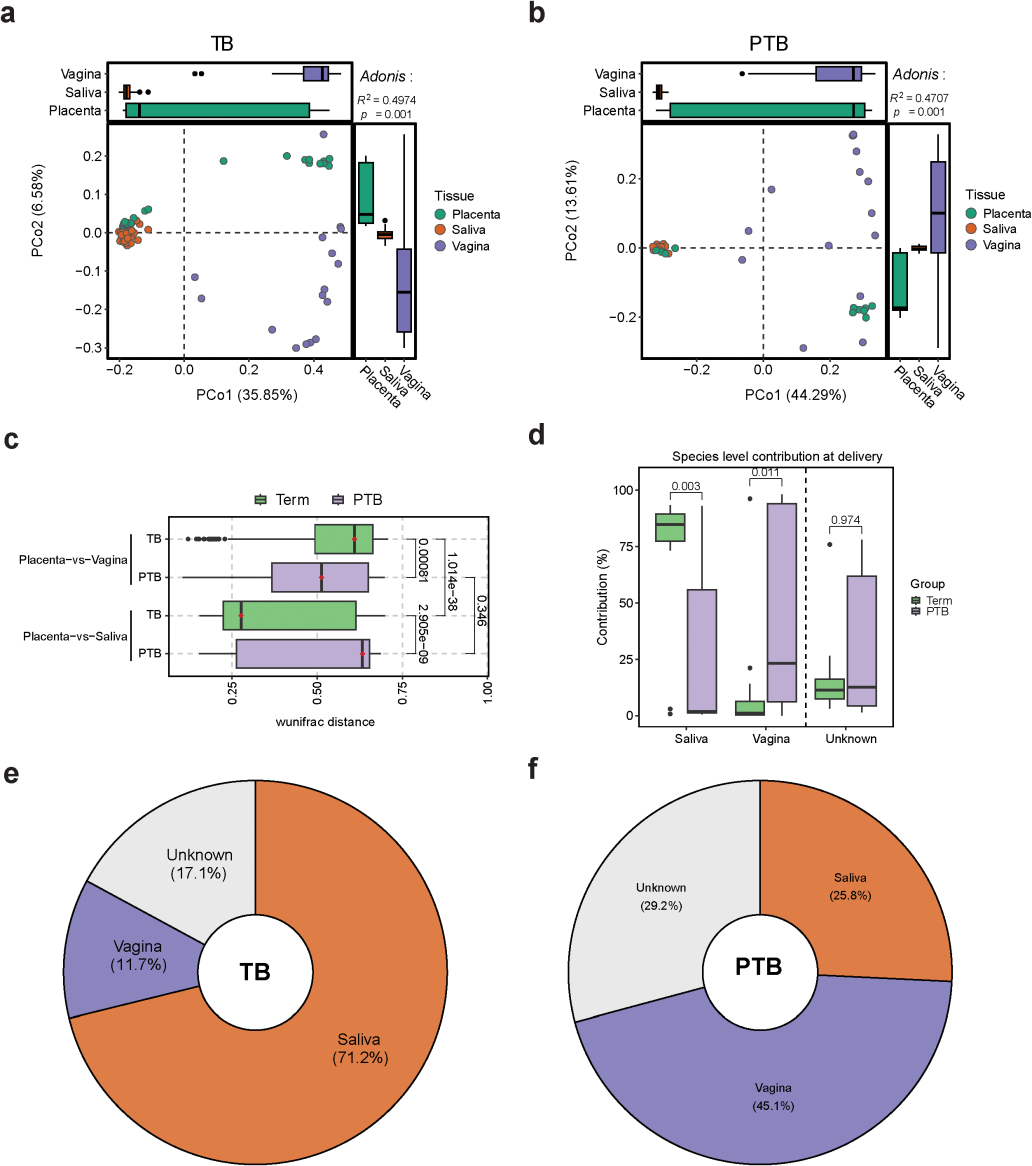
Comparing Oral, Vaginal, and Placental Microbiomes During Pregnancy **(A, B)** Bray-Curtis based PCoA plots showing the distribution of the microbiome composition in **(A)** TB samples and **(B)** PTB samples by body site. Adonis test *p*-values are shown in the top-right corners (999 permutations). **(C)** Box plot shows pairwise-weighted UniFrac distances between placenta, vagina, and saliva samples collected from TB and PTB women. (Green: TB samples; purple: PTB samples). All *p*-values were calculated using the Wilcoxon test. **(D)** Box plot showing the % of microbial sharing between placenta, saliva, and vagina, at delivery. **(E, F)** Pie chart representing the sources of the placental microbiome calculated using FEAST.

Our PCoA analysis revealed that the placental and oral microbiomes clustered closely together in the TB group (**Figure 3A**), whereas the placental and vaginal microbiomes were closer in the PTB group (**Figure 3B**). Comparative analysis using the weighted UniFrac distance revealed that, in PTB cases, the placental microbiome bears greater similarity to the vaginal microbiome than to the salivary microbiome (**Figure 3C**). Conversely, in the TB group, the placental microbiome is more similar to the oral microbiome (**Figure 3C**).

Overall, after analyzing the contribution of the oral and vaginal bacteria to the placental microbiome, we found that the oral microbiome had more contribution in the TB group, whereas more vaginal bacteria contributed to the placental microbiome composition in the PTB subjects (**Figure 3D**). Interestingly, our results showed that the placenta shared more species with the oral cavity than the vagina in the TB group. This provides further evidence in support for the existence of an oral-placental microbial sharing during term pregnancy.

Next, we calculated the percentage of vaginal and oral microbiome contribution to the placental microbiome (**Figures 3E, F**). Our data showed that in full term pregnancy, around 71% of the placental microbiome originates from the oral cavity, 12% from the vaginal environment and the rest from other sources (**Figure 3E**). During PTB, around 45% of the placental microbiome appear to be derived from the vaginal microbiome, with less contribution from the oral cavity (**Figure 3F**).

## Discussion

Pregnancy is a unique physiological state characterized by temporary changes in the women’s physical structure, hormone levels, metabolism, immunity, and microbiome composition (Kandan et al., 2011; Nuriel-Ohayon et al., 2016). In this study, we aimed to investigate the origin of the placental microbiome and the interrelationship between the various microbiomes in pregnant women who delivered at term and compare it to those who experienced preterm birth in a low resource setting. We conducted a case-control study, using our prospective MSP cohort, and assessed the multi-site microbiome composition in 18 PTB and 36 matched TB subjects.

Our results showed that the top phyla were concordant with previously reported oral microbiome compositions during pregnancy in TB and PTB groups (Zarco et al., 2012; Chen and Jiang, 2014; Costalonga and Herzberg, 2014; Cobb et al., 2017; Jang et al., 2021; Vidmar Simic et al., 2023). Many studies have reported a positive correlation between periodontal disease, oral pathogens and PTB (Ressler-Maerlender et al., 2005; Silva de Araujo Figueiredo et al., 2017; Saadaoui et al., 2021), this was also supported by our results showing that many species, such as *Treponema maltophilum* (Wyss et al., 1996), *Leptotrichia* (Ortiz et al., 2022), *Alloprevotella* (Kononen et al., 2022), and *Prevotella enoeca* increased in abundance in PTB subjects. *Prevotella* sp. *Alloprevotella*, *Mollicutes* and *Prevotella enoeca* increased in abundance, both at T2 and delivery, when we compared TB and PTB subjects, indicating their potential use as biomarkers for early detection of pregnant women with a higher PTB risk. More validation work is needed to confirm our findings.

To rule out the possibility of bacterial contamination from membrane rupture, we excluded samples from subjects who experienced prolonged rupture of membranes (≥18 hours), prior to analyzing the placenta microbiome data. Consistent with previous studies, our data showed an increase in *Ureaplasma urealyticum* and other *Ureaplasma species* in placenta samples collected from PTB compared to TB subjects (Kundsin et al., 1984; Kundsin et al., 1996; Olomu et al., 2009; Padmini et al., 2011; Aydogan et al., 2014; Suzuki et al., 2018). We hypothesize that those *Ureaplasma* species may originate from the vaginal cavity, which, in uncomplicated term pregnancies, is typically dominated by *Lactobacillus species* (Ravel et al., 2011; Fettweis et al., 2014; MacIntyre et al., 2015; Fettweis et al., 2019; Tabatabaei et al., 2019), whereas, *Gardnerella vaginalis*, *Ureaplasma species*, and other anaerobic bacteria have been linked to negative pregnancy outcomes (Breugelmans et al., 2010; Payne et al., 2016; Rittenschober-Bohm et al., 2018; Rittenschober-Bohm et al., 2019).

Researchers continue to investigate the origins of the placental microbiome, and they have proposed several hypotheses. For example, one hypothesis suggests that the placental microbiome may have its origins from the oral microbiome, while another contends that the vaginal microbiome may also have a role in the development of the placental microbiome by facilitating the ascent of diverse bacteria through the vaginal canal (Cao et al., 2014; Li et al., 2024). As far as we are aware, our study was the first to assess the interrelationships between oral, vaginal, and placental microbiomes collected from the same subjects and shed the light on the major differences in uncomplicated term pregnancies and PTB. Our data suggest that the placental microbiome was associated with the microbiome of the oral and the vaginal ecosystems. Around 17-29% of the placental microbiome appear to originate from unknown sources, this can include other microbial sites, environmental bacterial or potential contamination (Weiss et al., 2014; Li et al., 2024), which was not ruled out in this study.

Interestingly, our analysis showed that the placental microbiome showed a higher similarity to the oral microbiome, especially at the species level in subjects with uncomplicated term pregnancies. This is consistent with previous studies that reported a higher similarity between the microbiome of the placenta and oral cavity in uncomplicated term pregnancies (Aagaard et al., 2014; Gomez-Arango et al., 2017). This suggests that the oral microbiome is related to the placental microbiome in term pregnancy. Previous studies have indicated that oral disease is associated with adverse pregnancy outcomes, including premature birth (Moore et al., 2004), preeclampsia (Boggess et al., 2003), and miscarriages (Farrell et al., 2006). Larger studies investigating the association between integrated oral care and pregnancy outcomes are needed.

On the other hand, in PTB subjects, the placental microbiome exhibited a closer resemblance to the vaginal microbiome, this highlights the potential role of the vaginal microbiome in influencing placental microbial composition in PTB. This aligns with our previous findings (Kumar M et al., 2020) and other studies indicating that vaginal dysbiosis, is associated with PTB (Ahrodia et al., 2022). The transfer of microorganisms from the vaginal environment to the placenta could potentially trigger inflammatory responses that contribute to preterm labor and birth (Cotch et al., 1997; Stout et al., 2017; Tabatabaei et al., 2019; Bayar et al., 2020; Dunlop et al., 2021; Daskalakis et al., 2023).

Vaginal dysbiosis, characterized by a decrease in *Lactobacillus species* levels and an increase in microbial diversity, can lead to several pregnancy complications, while maintaining a healthy vaginal microbiome may reduce the risk of PTB (Janssen et al., 2022). More studies are needed to evaluate the efficacy and safety of the use of oral or vaginal probiotics in pregnant subjects.

In our analysis, we observed a higher dysbiosis score in saliva, vaginal and placental samples from PTB women compared to TB women. Consistent with previous studies (Gomez de Aguero et al., 2016; Fettweis et al., 2019; Yin et al., 2021), this finding indicates that an imbalance in the microbial composition is associated with PTB. These results highlighted the importance of maintaining microbial balance to maintain a healthy pregnancy.

The strength of our study includes frequent sample collection from diverse body sites of participants and comprehensive data collection at multiple time points throughout pregnancy. However, our study also has limitations. Our study mainly included participants from the Burman and Karen ethnicity, limiting the generalizability across other ethnic groups. The fact that not all samples were collected at the same time is another limiting factor. Our findings are also limited by the challenges of studying low-biomass microbiomes, such as the placenta, which are prone to contamination during sample collection, DNA extraction, and sequencing. In this study, our negative controls were not sequenced, and the risk of contamination was mainly assessed using computational methods.

Using 16S rRNA gene sequencing, we identified distinct microbial profiles in the oral and placental microbiomes of women who experienced PTB compared to those who delivered at term. Notably, the higher levels of *Treponema maltophilum* in the oral microbiome during the first trimester in PTB cases suggest its potential as an early biomarker for preterm risk. Our findings support that multiple maternal microbiomes play a role in shaping the composition of the placental microbiome. While placental microbial communities share more OTUs with the maternal oral microbiome than with the vaginal microbiome during term pregnancies, a greater sharing between the vaginal and placental microbiomes becomes apparent in preterm birth. Further investigation is needed to determine whether manipulating the oral or vaginal microbiome can influence the placental microbiome and affect pregnancy outcomes.

## Data Availability

The datasets presented in the study is available at Sequence Read Archive (SRA) repository, accession number PRJNA1153346.

## Supplementary material

Supplementary Figure 1 Overview of sample collection. Dot plots showing the number samples used (x-axis) and timepoints (y-axis) for TB **(A)** and PTB **(B)** groups. The size of the dots is proportionate to the number of samples. Dots are colored to indicate a timepoint. Trimester (T), delivery (D), TB (Term birth), PTB (Preterm birth).

Supplementary Figure 2 Diversity metric of salivary and placental species during pregnancy. **(A)** Box plots of different alpha-diversity measures comparing richness metrics (number of observed taxa, Chao1, ACE; left) and diversity metrics (Shannon, Simpson, and Pielou; right) of saliva samples from PTB and TB groups at T2 and delivery. Blue: PTB samples; orange: TB samples. The numbers above the box plots represent the *p* value. **(B)** Principal coordinate analysis (PCoA) plot showing the distribution of samples from PTB and TB groups. **(C)** PCoA plot showing the distribution of the samples from PTB and TB groups at different time points. Adonis test *p*-values are shown in the top-right corners (999 permutations). The top and right boxplots show the distribution of the samples on the PCoA1 and PCoA2 axis, respectively. **(D)** Box plots of different alpha-diversity measures comparing richness metrics (number of observed taxa, Chao1, ACE; left) and diversity metrics (Shannon, Simpson, and Pielou; right) of placental samples from PTB and TB groups at delivery. Blue: PTB samples; orange: TB samples. The numbers above the box plots represent the *p* value. **(E)** PCoA plot shows the distribution of placental samples from PTB and TB groups at delivery. Adonis test *p*-values are shown on the top-right corner (999 permutations). The top and right boxplots show the distribution of samples on the PCoA1 and PCoA2 axis, respectively.

Supplementary Figure 3
**(A, B)** Electrophoresis gel of water control **(C)** and placental samples collected from TB and PTB subjects.

Supplementary Figure 4 Dysbiosis score per-tissue during pregnancy. **(A-C)** Boxplot plot showing the distribution and the statistical significance between TB and PTB and TB samples in Vagina, Saliva and Placenta respectively. The central line represents median values, while the box edges represent the interquartile range (IQR). Wiskers extend to 1.5 times the IQR. Statistical significance was estimated using Wilcoxon rank-sum test.

Supplementary Figure 5 Relationship between dysbiosis score and microbial diversity during pregnancy. **(A-C)** Scatter plots showing the correlation between the dysbiosis score (x-axis) and the number of uniquely observed species (y-axis) in vagina, saliva and placenta respectively in samples collected during the first, second and third trimesters (T1, T2, T3), and at delivery **(D)**. Pearson correlation coefficients (R) and correlation test *p*-values are indicated in each plot. Significant correlations are shown in bold font.
