# Supplementary figures and images for: Exploring the composition of placental microbiome and its potential origin in preterm birth
Source: Front Cell Infect Microbiol. 2025 Jan 16;14:1486409. doi: 10.3389/fcimb.2024.1486409 (PMC11779731; doi:10.3389/fcimb.2024.1486409)

**a**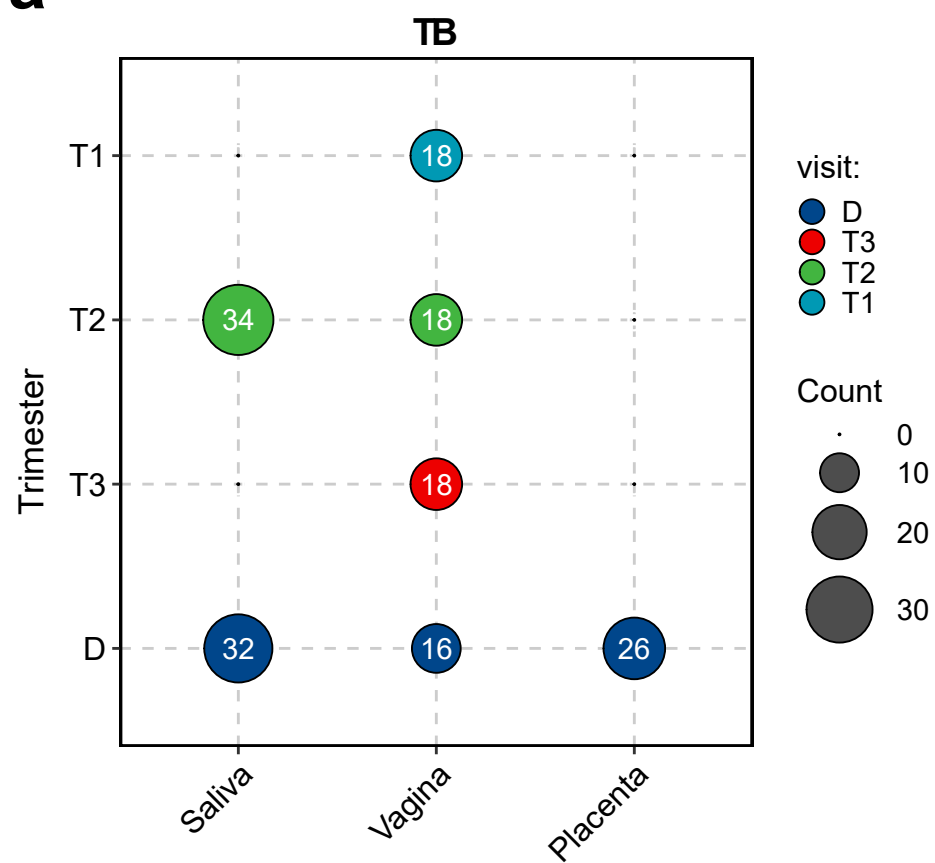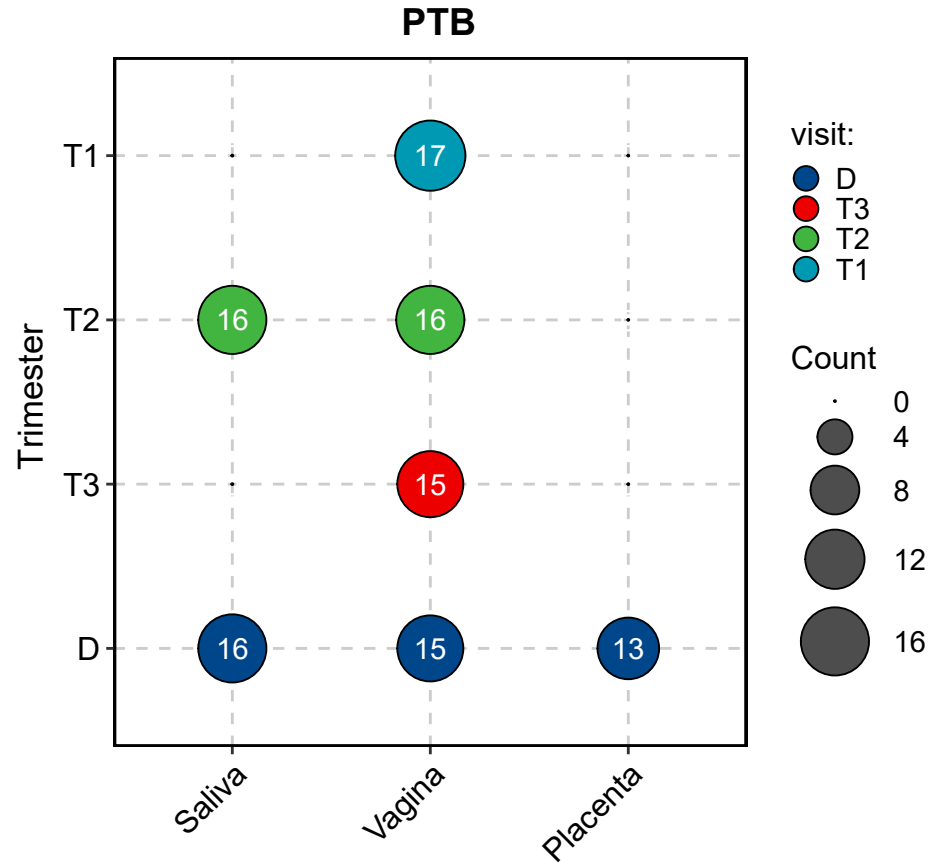

Supplement: Supplementary Figure 1 — Overview of sample collection. Dot plots showing the number samples used (x-axis) and timepoints (y-axis) for TB (A) and PTB (B) groups. The size of the dots is proportionate to the number of samples. Dots are colored to indicate a timepoint. Trimester (T), delivery (D), TB (Term birth), PTB (Preterm birth). [file DataSheet1.pdf]

a

Saliva

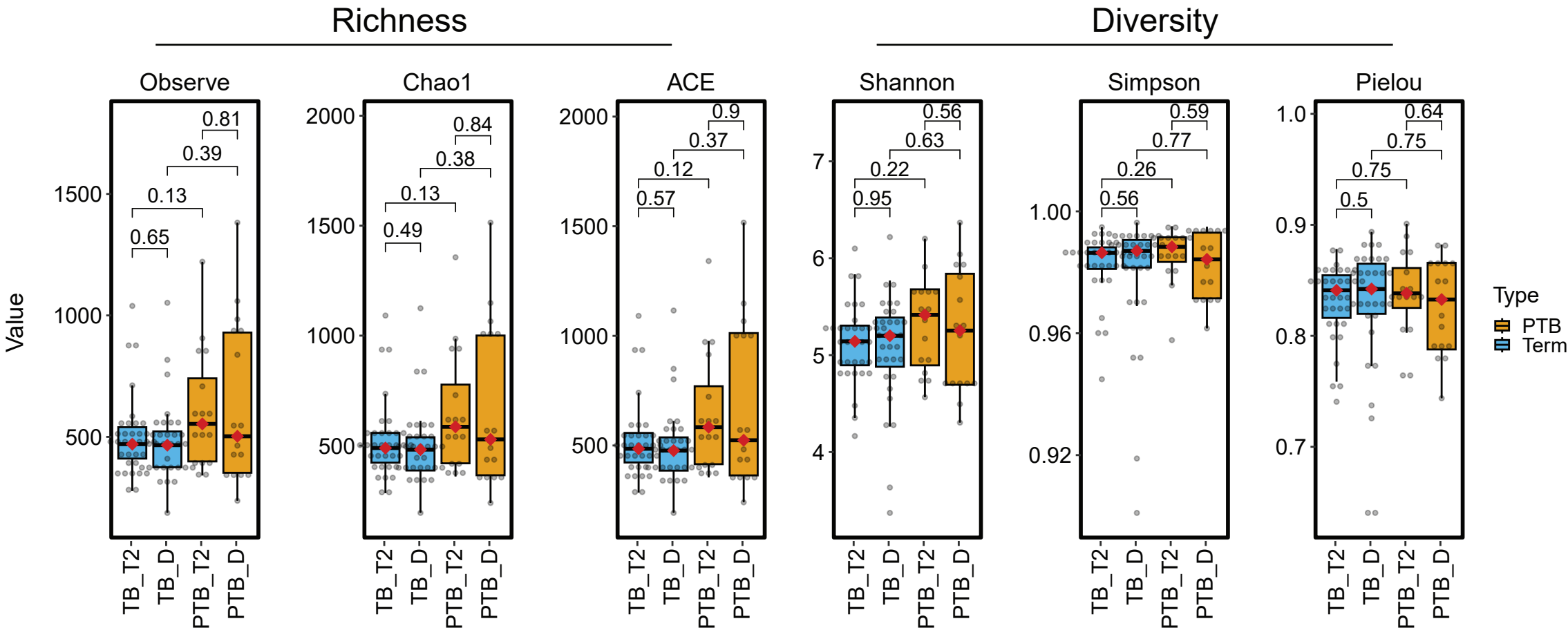

b

Saliva

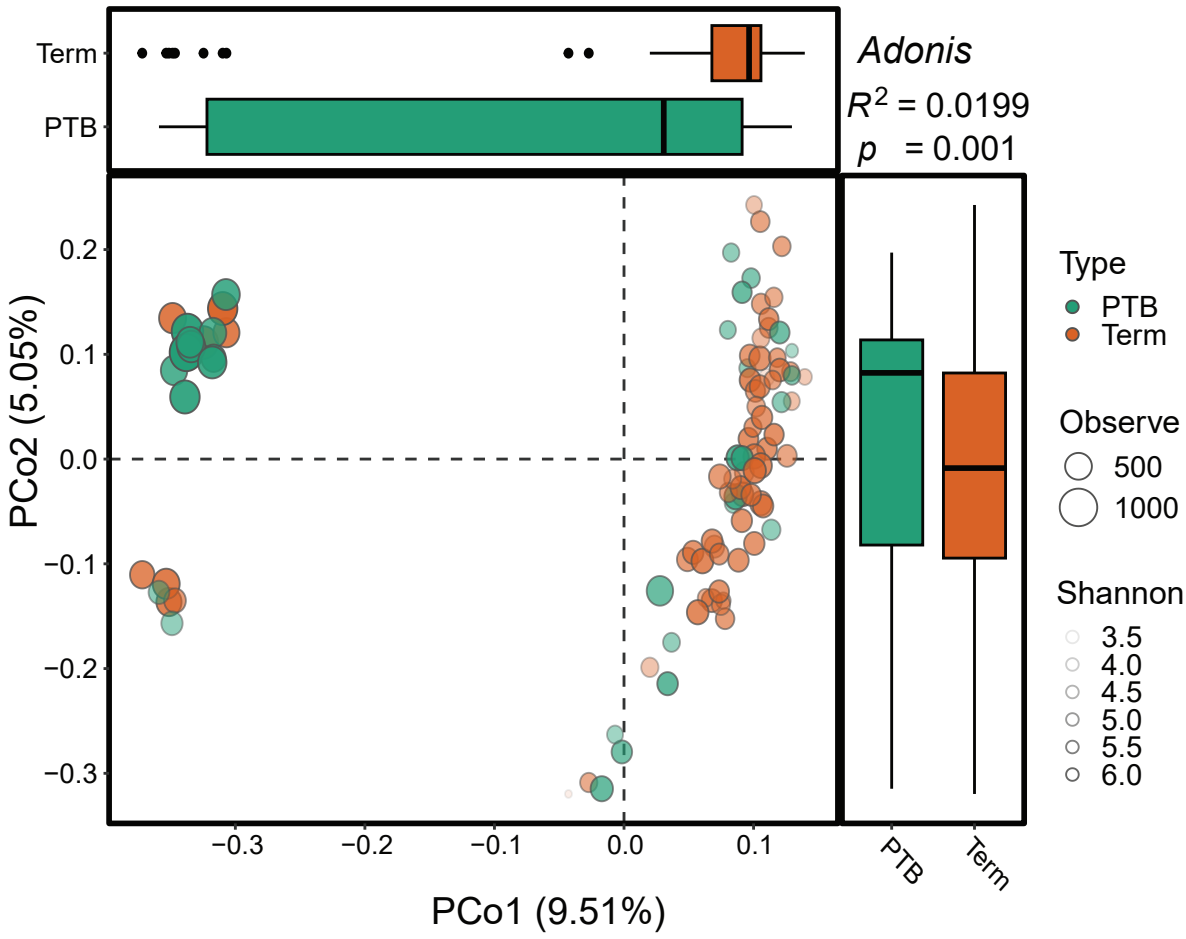

c

Saliva

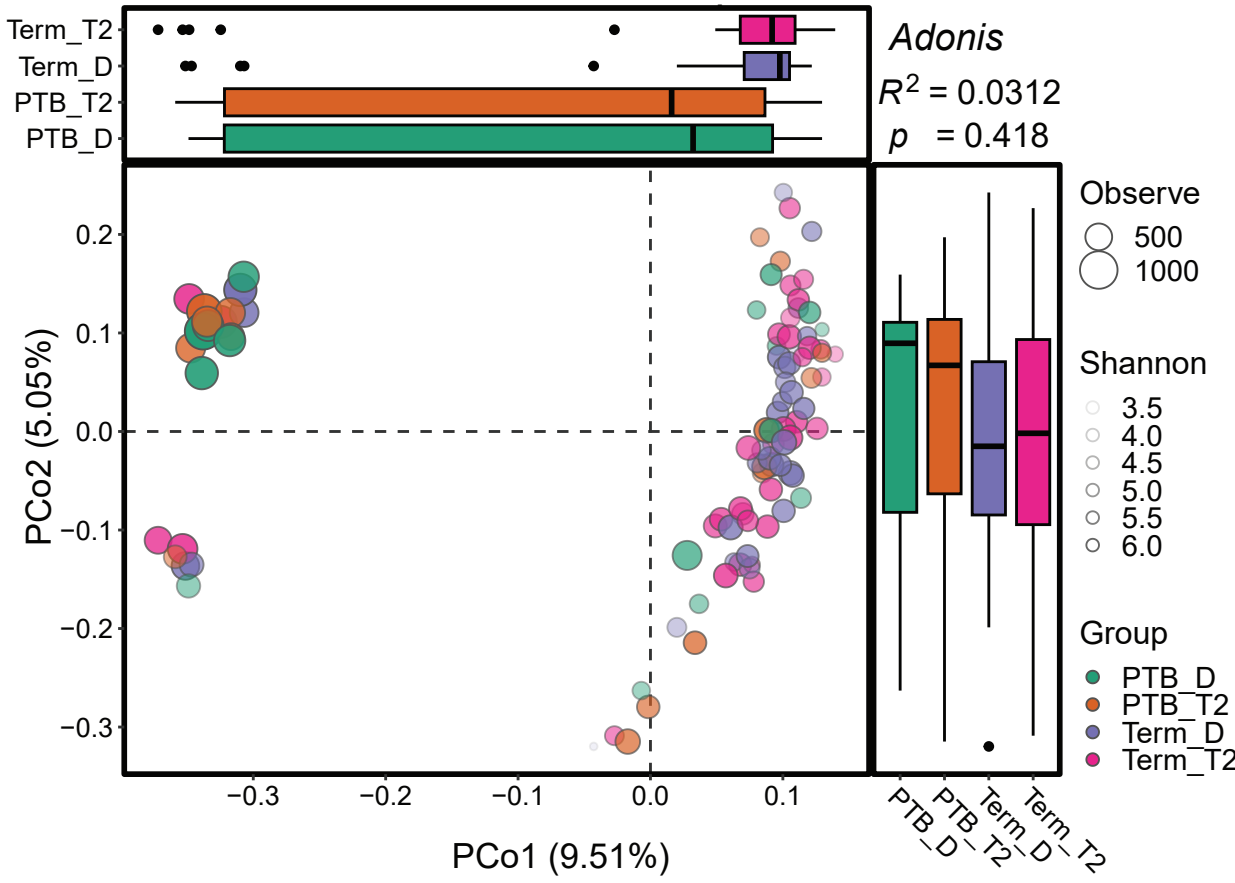

d

Placenta

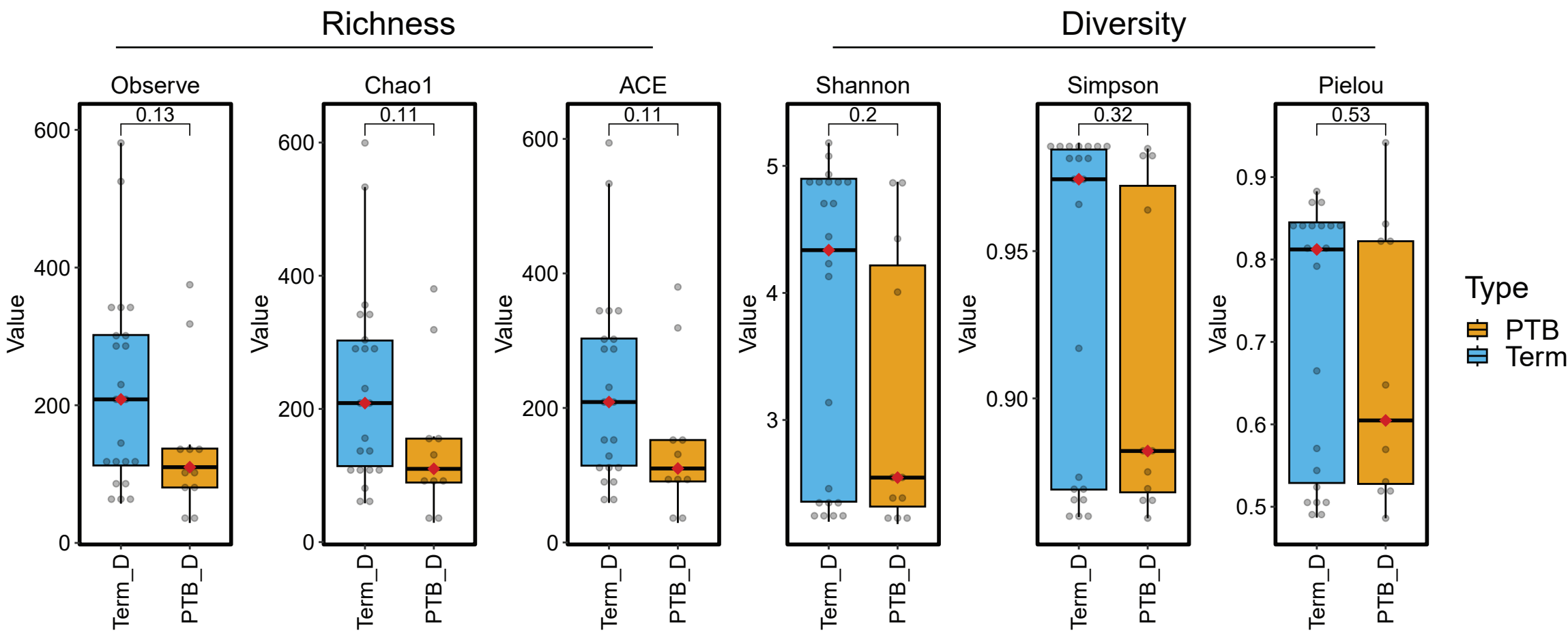

e

Placenta

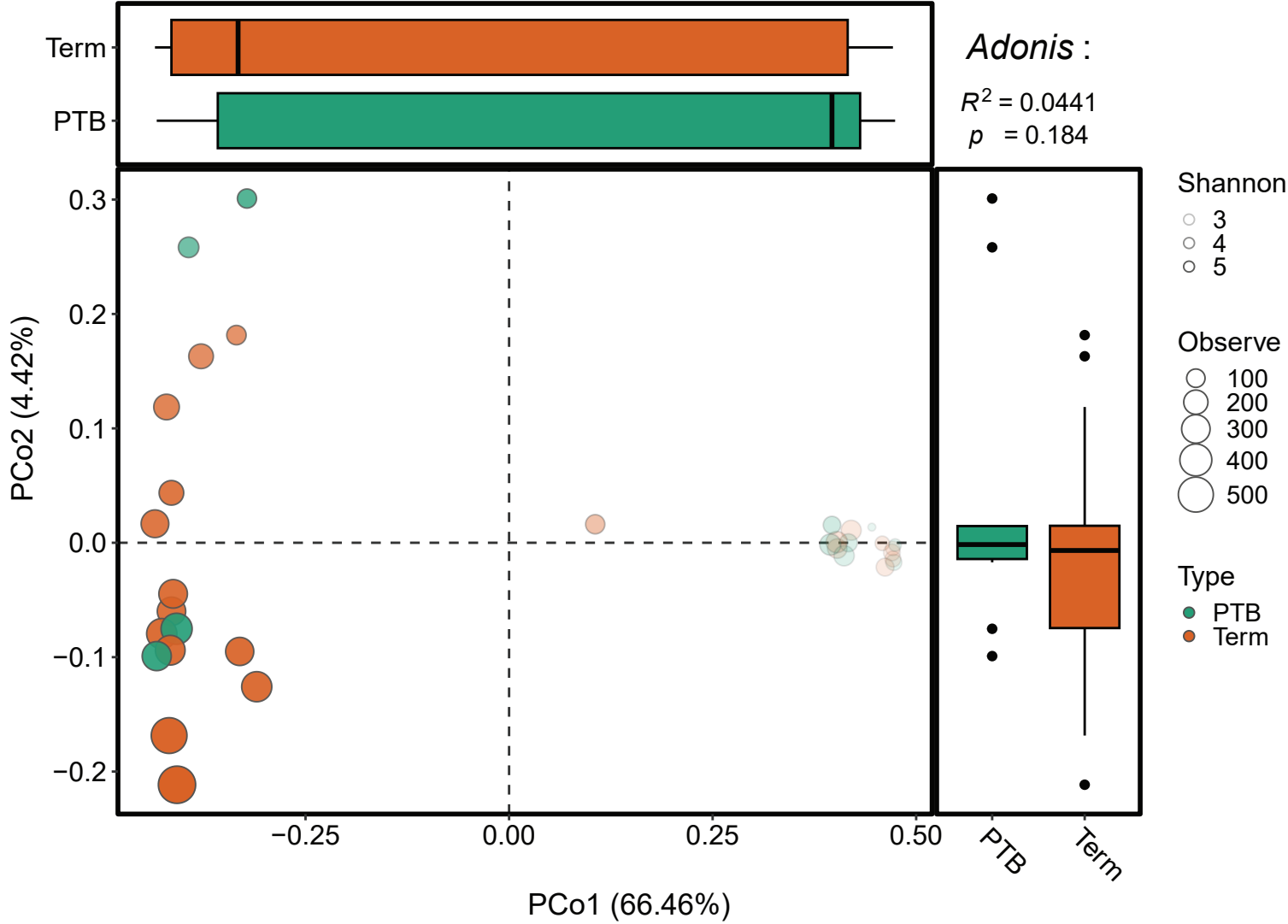

Supplement: Supplementary Figure 2 — Diversity metric of salivary and placental species during pregnancy. (A) Box plots of different alpha-diversity measures comparing richness metrics (number of observed taxa, Chao1, ACE; left) and diversity metrics (Shannon, Simpson, and Pielou; right) of saliva samples from PTB and TB groups at T2 and delivery. Blue: PTB samples; orange: TB samples. The numbers above the box plots represent the p value. (B) Principal coordinate analysis (PCoA) plot showing the distribution of samples from PTB and TB groups. (C) PCoA plot showing the distribution of the samples from PTB and TB groups at different time points. Adonis test p-values are shown in the top-right corners (999 permutations). The top and right boxplots show the distribution of the samples on the PCoA1 and PCoA2 axis, respectively. (D) Box plots of different alpha-diversity measures comparing richness metrics (number of observed taxa, Chao1, ACE; left) and diversity metrics (Shannon, Simpson, and Pielou; right) of placental samples from PTB and TB groups at delivery. Blue: PTB samples; orange: TB samples. The numbers above the box plots represent the p value. (E) PCoA plot shows the distribution of placental samples from PTB and TB groups at delivery. Adonis test p-values are shown on the top-right corner (999 permutations). The top and right boxplots show the distribution of samples on the PCoA1 and PCoA2 axis, respectively. [file DataSheet2.pdf]

**a**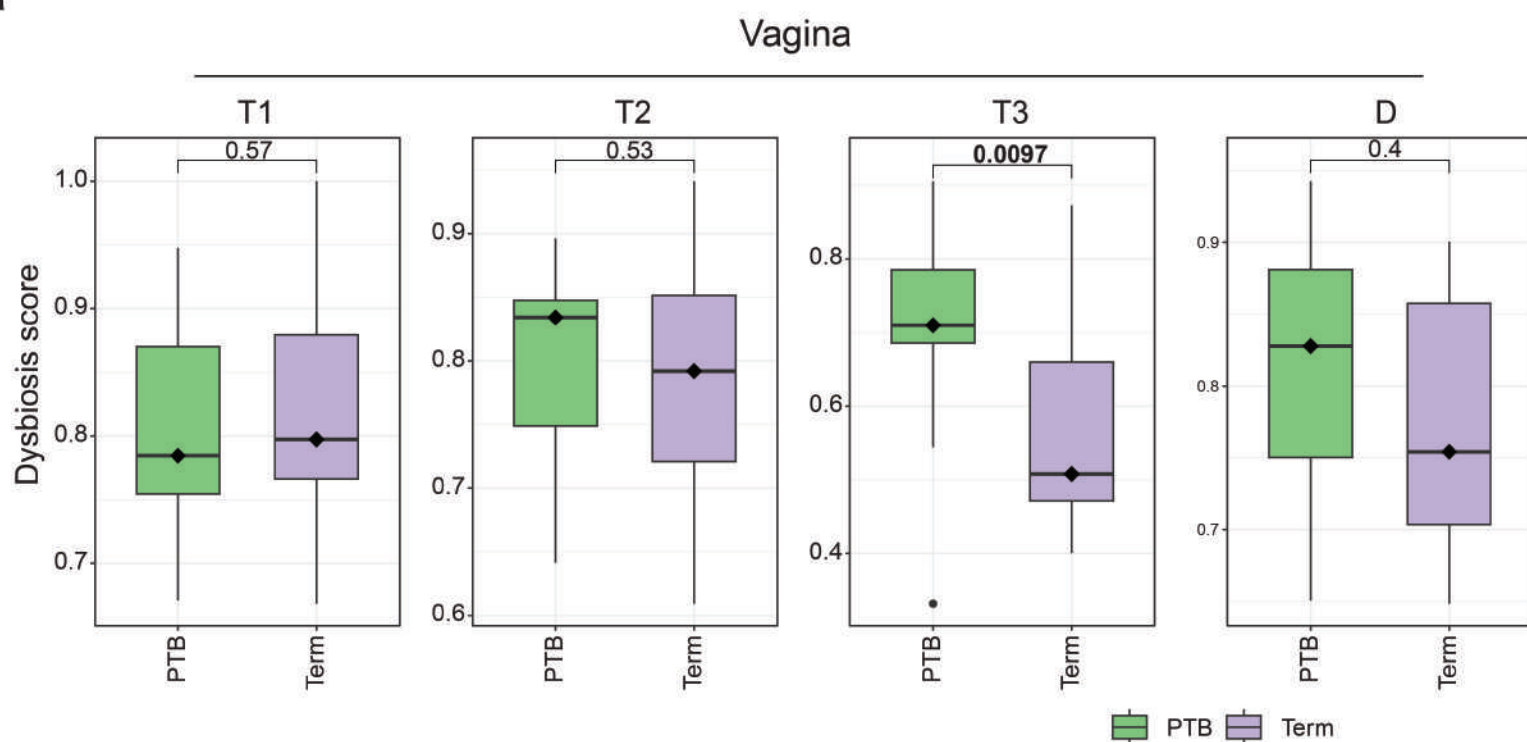**b**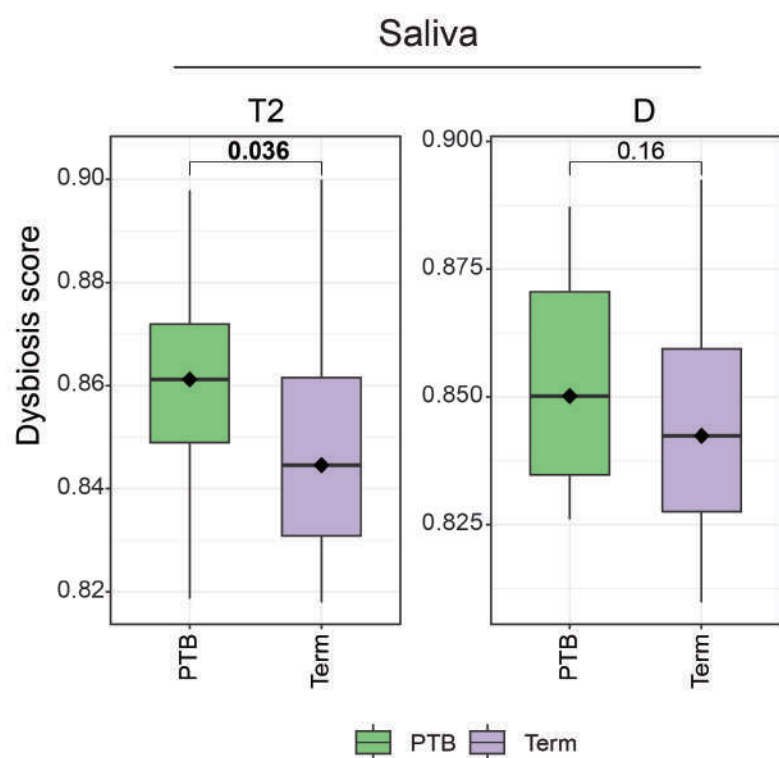**c**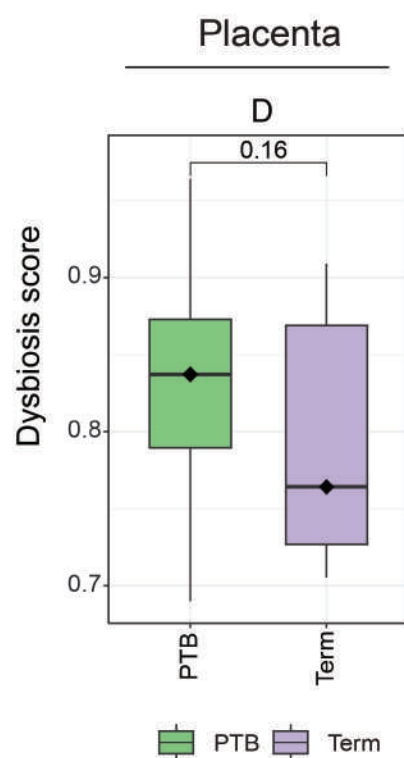

Supplement: Supplementary Figure 3 — (A, B) Electrophoresis gel of water control (C) and placental samples collected from TB and PTB subjects. [file DataSheet3.pdf]

a

Vagina\_T1

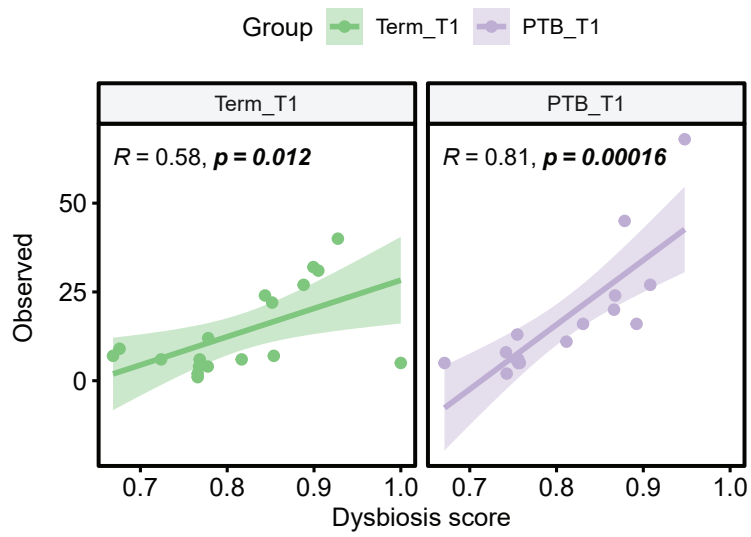

Vagina\_T2

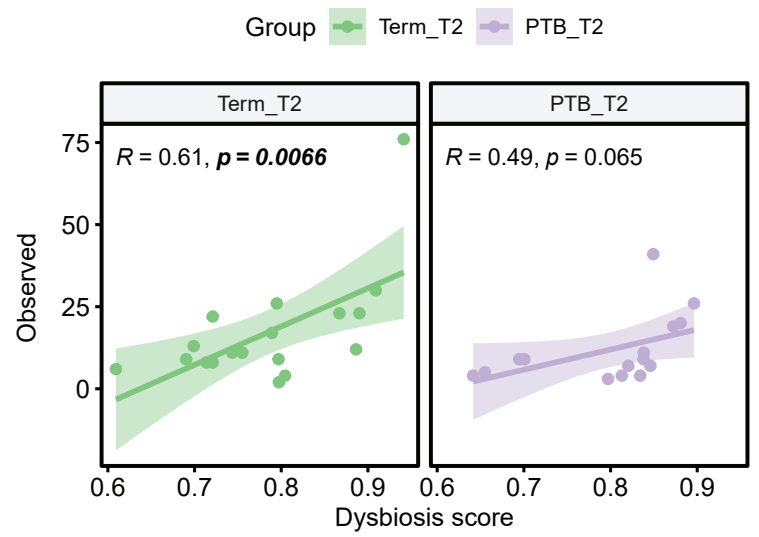

Vagina\_T3

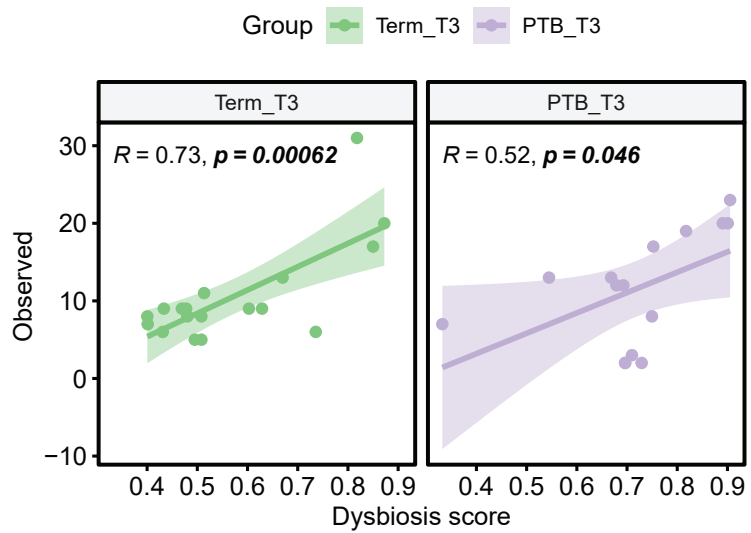

Vagina\_D

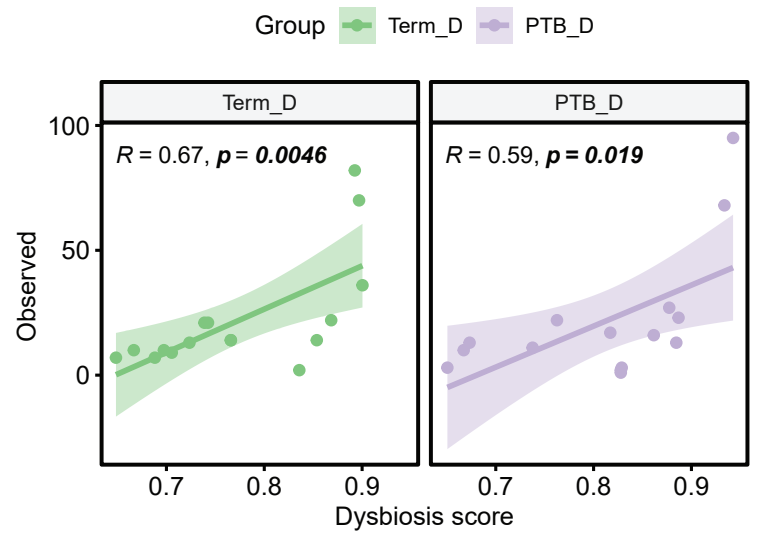

b

Saliva\_T2

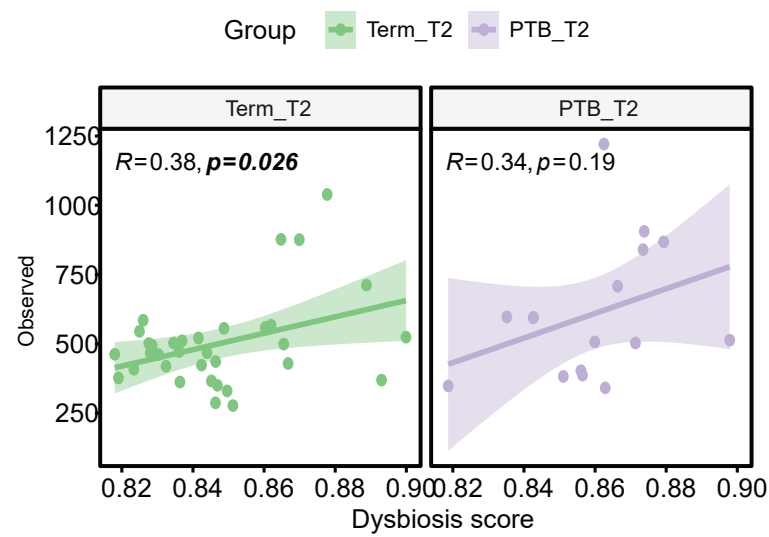

Saliva\_D

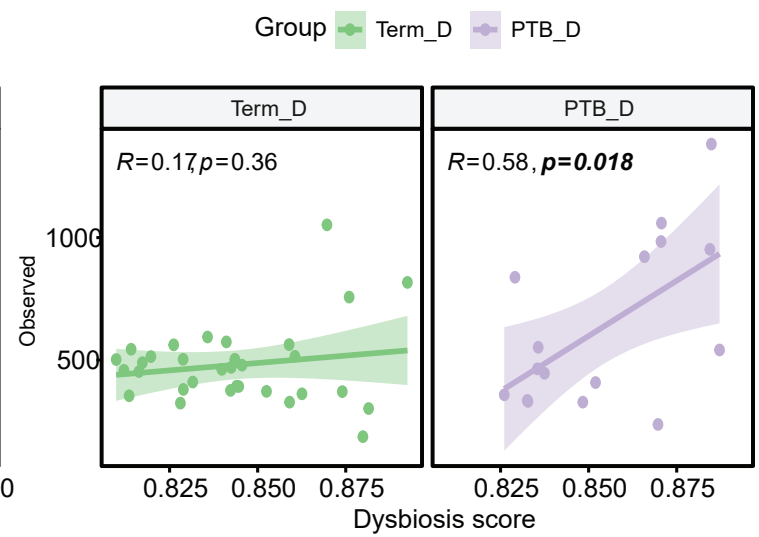

c

Placenta\_D

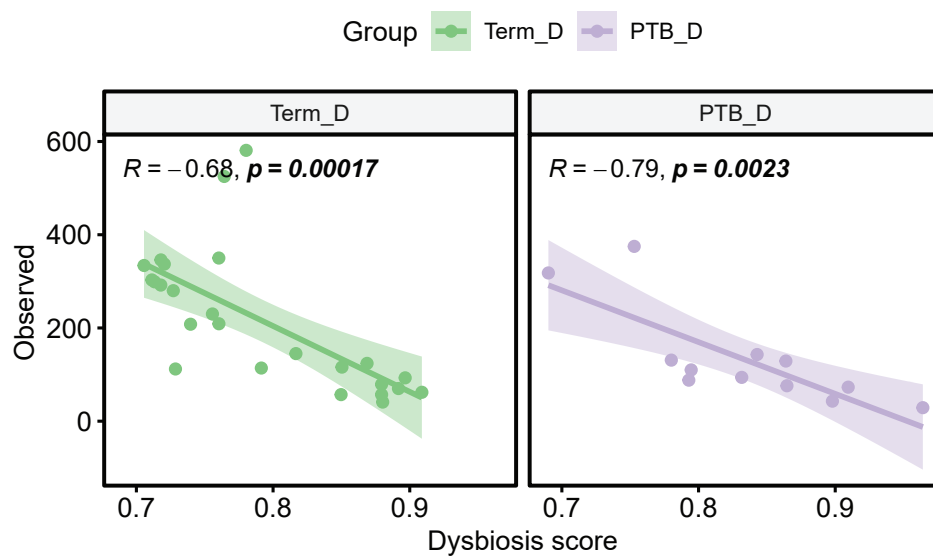

Supplement: Supplementary Figure 4 — Dysbiosis score per-tissue during pregnancy. (A-C) Boxplot plot showing the distribution and the statistical significance between TB and PTB and TB samples in Vagina, Saliva and Placenta respectively. The central line represents median values, while the box edges represent the interquartile range (IQR). Wiskers extend to 1.5 times the IQR. Statistical significance was estimated using Wilcoxon rank-sum test. [file DataSheet4.pdf]

a

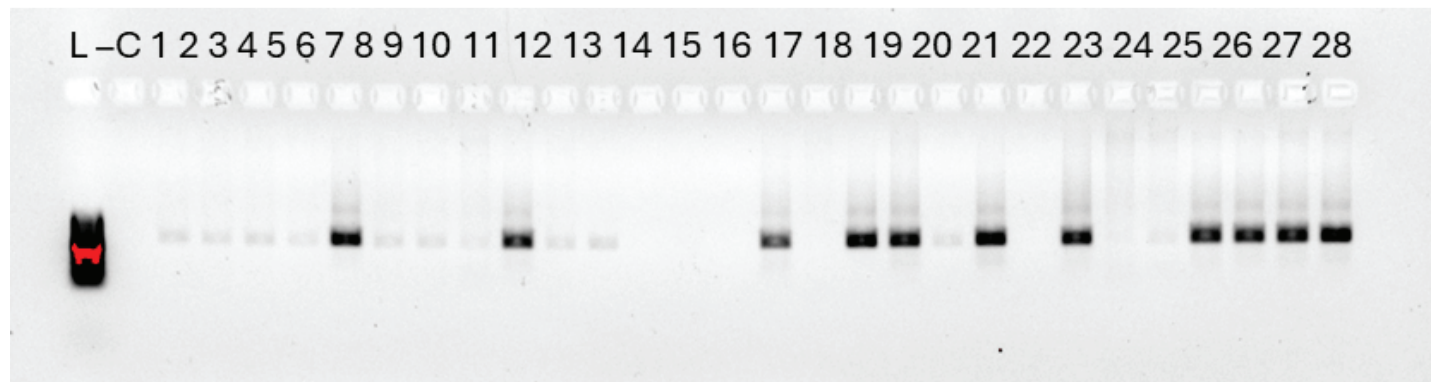

b

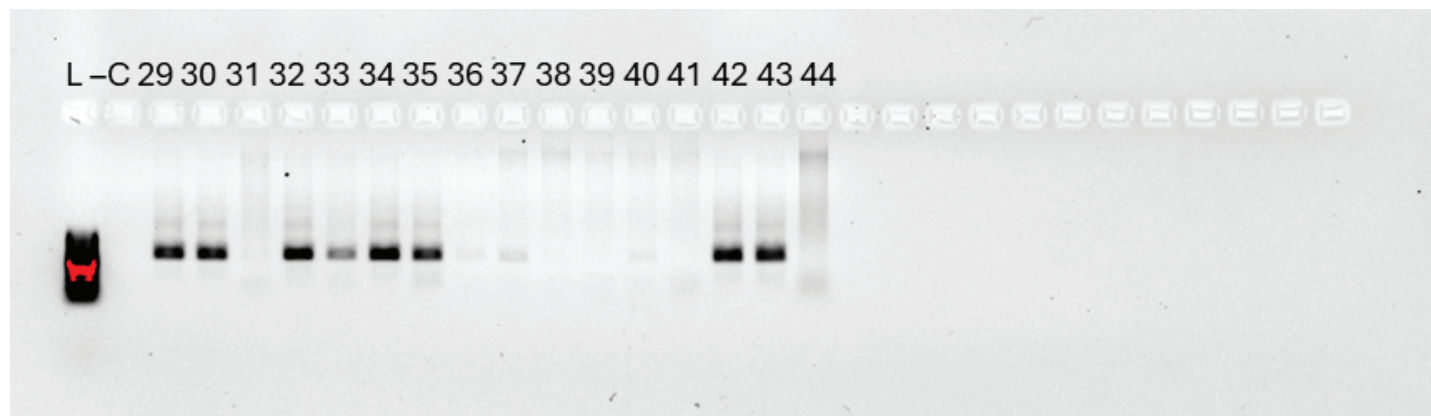

Supplement: Supplementary Figure 5 — Relationship between dysbiosis score and microbial diversity during pregnancy. (A-C) Scatter plots showing the correlation between the dysbiosis score (x-axis) and the number of uniquely observed species (y-axis) in vagina, saliva and placenta respectively in samples collected during the first, second and third trimesters (T1, T2, T3), and at delivery (D). Pearson correlation coefficients (R) and correlation test p-values are indicated in each plot. Significant correlations are shown in bold font. [file DataSheet5.pdf]
