# Supplementary material for: Exploring the composition of placental microbiome and its potential origin in preterm birth
Source: Front Cell Infect Microbiol. 2025 Jan 16;14:1486409. doi: 10.3389/fcimb.2024.1486409 (PMC11779731; doi:10.3389/fcimb.2024.1486409)
Supplement: Supplementary file 6 [file Table1.docx]

**Supplementary Table 1: Description of the cohort**

|  | **TB (n=36)** | | **PTB (n=18)** | |  |
| --- | --- | --- | --- | --- | --- |
|  | **Median** | **IQR [Q25, Q75]** | **Median** | **IQR [Q25, Q75]** | **P-value*^1^*** |
| **Age at enrolment (years)** | 24.0 | [21.0, 27.0] | 21.5 | [20.0, 24.5] | 0.26 |
| **Height at enrolment (cm)** | 151.6 | [149.2, 155.4] | 154.2 | [150.5, 155.6] | 0.27 |
| **Weight at enrolment (kilograms)** | 48.0 | [44.3, 55.3] | 48.0 | [42.3, 48.9] | 0.33 |
| **BMI at enrolment** | 20.9 | [19.3, 23.4] | 20.1 | [18.2, 20.4] | 0.15 |
| **Outcome EGA (days)** | 276.5 | [269.8, 283.0] | 253.5 | [242.0, 254.8] | <0.001 |
| **Birth weight (grams)** | 3,060.0 | [2,907.5, 3,310.0] | 2,265.0 | [1,980.0, 2,440.0] | <0.001 |
| **Length of rupture of membranes (hours)** | 1.7 | [0.3, 21.3] | 2.7 | [0.2, 60.5] | 0.62 |
| **Head circumference (cm)** | 33.1 | [32.4, 33.7] | 30.4 | [30.0, 31.5] | <0.001 |
| *^1^*Mann-Whitney U Test | | | | | |
| EGA: Estimated Gestational Age; IQR: Interquartile Range; PTB: Preterm Birth; TB: Term Birth | | | | | |
